# Socio-demographic patterns of disability among older adult populations of low-income and middle-income countries: results from World Health Survey

**DOI:** 10.1007/s00038-015-0742-3

**Published:** 2015-11-04

**Authors:** Ahmad Reza Hosseinpoor, Nicole Bergen, Nenad Kostanjsek, Paul Kowal, Alana Officer, Somnath Chatterji

**Affiliations:** Department of Health Statistics and Information Systems, World Health Organization, 20, Avenue Appia, 1211 Geneva 27, Switzerland; Department of Ageing and Life Course, World Health Organization, 20, Avenue Appia, 1211 Geneva 27, Switzerland

**Keywords:** Disabled persons, Developing countries, Aged, Socioeconomic factors, Prevalence

## Abstract

**Objective:**

Our objective was to quantify disability prevalence among older adults of low- and middle-income countries, and measure socio-demographic distribution of disability.

**Methods:**

World Health Survey data included 53,447 adults aged 50 or older from 43 low- and middle-income countries. Disability was a binary classification, based on a composite score derived from self-reported functional difficulties. Socio-demographic variables included sex, age, marital status, area of residence, education level, and household economic status. A multivariate Poisson regression model with robust variance was used to assess associations between disability and socio-demographic variables.

**Results:**

Overall, 33.3 % (95 % CI 32.2–34.4 %) of older adults reported disability. Disability was 1.5 times more common in females, and was positively associated with increasing age. Divorced/separated/widowed respondents reported higher disability rates in all but one study country, and education and wealth levels were inversely associated with disability rates. Urban residence tended to be advantageous over rural. Country-level datasets showed disparate patterns.

**Conclusions:**

Effective approaches aimed at disability prevention and improved disability management are warranted, including the inclusion of equity considerations in monitoring and evaluation activities.

**Electronic supplementary material:**

The online version of this article (doi:10.1007/s00038-015-0742-3) contains supplementary material, which is available to authorized users.

## Introduction

Disabilities—significant impairments, activity limitations or participation restrictions that result from an interaction of a health condition with contextual factors (World Health Organization 2001)—directly affect more than one billion people worldwide (World Health Organization 2011). Each individual living with a disability represents a unique experience, shaped by their physiological condition as well as the social and physical environments where they live and work. Despite the diversity in disabling conditions, people living with disabilities face common barriers that prevent full participation in society. Disabling barriers stem from inadequate policies or standards, negative attitudes and prejudices, service deficiencies, inaccessible built environments, and a lack of evidence and data to inform effective policies and programs (World Health Organization 2011). People affected by disabilities are at an increased risk for poor health outcomes, lower education attainment, reduced employment and earning potential, living in poverty, and higher dependency on others (World Health Organization 2011).

Changing ideologies and the absence of a universal ‘gold standard’ measurement of disability have impeded efforts to enumerate, track and make international comparisons of disability prevalence (Barbotte et al. 2001). Over the last several decades, the perception of disability has shifted from a biomedical focus on individual deficiencies, to encompass contextual factors related to socio-cultural and political constructs (Imrie 2004). In 2006, the United Nations adopted the Convention on the Rights of Persons with Disabilities (CRPD), advancing disability as a human rights and development issue (United Nations 2006). The World Health Organization International Classification of Functioning, Disability and Health is built on the most internationally accepted framework of disability, a conceptualisation that incorporates medical and social models along with a right-based approach to disability (World Health Organization 2001). Recognizing disability as a global health issue, a human rights issue and a priority for development, in 2014 the World Health Organization endorsed the WHO Global Disability Action Plan 2014–2021: Better Health for all People with Disability, which has the overall goal of achieving health, wellbeing and human rights for persons with disabilities (World Health Assembly 2014).

Although disabilities affect people of all ages, genders, geographical regions, education levels and socioeconomic positions, some groups may be more likely to develop disabilities (Elwan 1999). Individuals and groups may differ in their ability to manage adverse health conditions (Commission on Social Determinants of Health 2008). Worldwide adult disability prevalence estimates from the World Health Survey (WHS) and Global Burden of Disease Study were calculated at 15.6 and 19.4 %, respectively, with higher prevalence in developing countries and in older age (World Health Organization 2008, 2011). Women have consistently reported higher rates of disability than men (Newman and Brach 2001), and a large body of research supports an inverse association between socioeconomic status and disability prevalence (Adamson et al. 2003; Ebrahim et al. 2004). These relationships, however, may not persist at all ages (Minkler et al. 2006), and may demonstrate variance according to the type of disability measure (Beydoun and Popkin 2005). Few studies have explored these patterns in low- and middle-income countries (LMICs). In addition, other demographic characteristics such as marital status and urban/rural place of residence variably correlated with disability measures in some settings (Beydoun and Popkin 2005; Kisioglu et al. 2003), although a lack of comparable international data precludes conclusive generalizations about the socio-demographic distribution of disability on a global scale.

As life expectancies increase and chronic conditions and injuries become more common, the tasks of describing disability trends and understanding how disabilities affect populations become increasingly relevant (Zarocostas 2011). Within the next decade the global population of older people will surpass that of children for the first time (United Nations Department of Economic and Social Affairs Population Division 2010), and the number of older adults living with a disability will rise substantially (Giles et al. 2003).

Cross-national comparisons of disabilities in older adults constitute valuable additions to the field of disability epidemiology (Guralnik 2005), helping to establish preventive health priorities and ensure that support services and interventions are directed to areas of greatest need (World Health Organization Regional Office for the Western Pacific 2003). Using WHS data from adults over 50, our objective was to quantify the prevalence of disability across a large sample of LMICs, and measure the distribution of disability by selected socio-demographic factors: sex, age, marital status, education, household wealth and urban/rural place of residence. Given the paucity of comparable data from LMICs, the results of this study will serve as a benchmark for measuring and tracking disabilities and the socio-demographic distribution of disabilities in this understudied population.

## Methods

### Study population

The WHS is a valid, reliable and comparable source of international health data, describing characteristics of individual health and health systems (Ustun et al. 2003). The 2002–2004 WHS compiled data of adults aged 18 and older in 70 countries across all world regions. Household and individual questionnaires were used to gather data about socioeconomic status, demographics and self-reported domains of health. Surveys were probabilistically selected, with a non-zero chance of inclusion assigned to all individuals. Post-stratification corrections were made to sampling weights to adjust for non-response and population distribution patterns, as represented by the United Nations Statistical Division (http://unstats.un.org/unsd/default.htm) (Moussavi et al. 2007).

Data from 48 LMICs were obtained from the WHS. Of these, five countries were excluded because data for relevant variables were missing for more than 25 % of respondents (four countries), or the sample size was too low (one country, with only 148 eligible respondents). The data included in these analyses consist of 53,447 respondents aged 50 and over from the remaining 43 countries (19 low-income countries and 24 middle-income countries, as classified by the World Bank Group). Online Resource Table 1 shows the sample size by country, including the rate of missing response. Samples are nationally representative except the following, which were conducted in geographically limited regions: China, Comoros, Congo, Côte d’Ivoire, India, and the Russian Federation. Response rates at the household level were over 70 % in all 43 countries except for Congo (64 %) and Czech Republic (24 %). Individual-level response rates were above 82 % (http://www.who.int/healthinfo/survey/en/index.html).

Informed consent was obtained in all surveys through a procedure approved by institutional ethics review boards. A standard consent form was read to the respondent in the respondent’s language. If the respondent agreed to participate in the survey and was literate, the form was provided to the respondent to read over and sign, and was countersigned by the interviewer. If the respondent was illiterate and gave consent to participate, the interviewer confirmed this consent and signed on the form that the respondent had read the form, understood the study, and agreed to participate (http://www.who.int/healthinfo/survey/instruments/en/index.html).

### Variables

A binary classification of disability was the dependent variable. Survey questions collected self-reported data about difficulties in functioning within eight health domains: affect, cognition, interpersonal activities, mobility, pain and discomfort, self-care, sleep and energy, and vision. Data were scored using item response theory, and a partial credit model was used to calculate a composite disability score, ranging from zero (absence of disability) to 100 (complete disability) (Wilson et al. 2006). A score of 40 or above was chosen as a threshold for significant disability in everyday life, and served as the cutoff point to be classified as having a disability (Hosseinpoor et al. 2012; World Health Organization 2011).

Independent variables were selected in accordance with findings presented by the Commission on Social Determinants of Health (2008), and included sex, age (expressed categorically as 50–59, 60–69, 70–79, or 80+ years), marital status (married/cohabiting, never married, or divorced/separated/widowed), area of residence (rural or urban), education level (less than primary school, primary/secondary school completed, or high school completed or above), and household economic status (expressed as quintiles). Household economic status was determined using a dichotomous hierarchical ordered probit model, based on ownership of selected assets and access to certain services (Gakidou et al. 2007; Ustun et al. 2003). The resulting index was divided into quintiles within each country. Pooled results for 43 LMICs represent combined wealth quintiles based on country-specific classification. The use of an asset- and service-based household wealth measure along with education level helped to capture a broader picture of socioeconomic position than a single indicator.

### Statistical analysis

Overall disability prevalence and the disability prevalence according to each independent variable were calculated for adults aged 50 or above in the pooled dataset, and for each of the 43 LMICs included in this study. We refer to this as the ‘crude prevalence’ of disability because data were not adjusted for any other factors.

Next, a multivariate Poisson regression model with robust variance was used to assess the adjusted associations between disability and each of the independent variables in the pooled dataset and to generate prevalence rate ratio values with 95 % confidence interval (CI) (‘adjusted associations’). This model provides more accurate estimates compared with logit models when the binary outcome has a high prevalence (Barros and Hirakata 2003).

All analyses were weighted to account for the individual country survey sample designs and allowances were made for non-independence within country clusters. Stata 11 was used in all analyses.

## Results

### Overall prevalence

The overall unadjusted prevalence of disability in the pooled sample was 33.3 % (95 % CI 32.2–34.4 %). Disability prevalence varied widely among countries, ranging from 10 % or less in Malaysia and Uruguay, to over 50 % in Comoros and Bangladesh (Table 1). In 33 of the 43 study countries at least one out of five older adults lived with a disability.

**Table 1:** Crude prevalence of disability among adults aged 50 years or above, World Health Survey, 2002–2004

Country	Estimate	95 % CI	
Bangladesh	52.2	48.2	56.3
Bosnia and Herzegovina	39.0	29.9	48.2
Brazil	32.6	29.5	35.7
Burkina Faso	29.2	23.7	34.8
Chad	38.5	33.5	43.5
China	13.8	8.2	19.4
Comoros	66.4	61.3	71.6
Congo	32.7	22.9	42.4
Cote d’Ivoire	34.4	28.5	40.2
Croatia	32.1	26.9	37.2
Czech Republic	29.3	22.6	36.0
Dominican Republic	18.2	14.6	21.7
Ecuador	27.6	22.9	32.3
Estonia	22.9	17.5	28.4
Ethiopia	28.2	24.4	31.9
Georgia	38.3	33.6	43.0
Ghana	26.1	22.8	29.3
India	44.1	40.8	47.3
Kazakhstan	36.2	28.5	43.9
Kenya	31.9	26.0	37.7
Latvia	38.6	32.7	44.5
Malawi	21.5	17.9	25.0
Malaysia	7.6	5.8	9.5
Mali	16.7	13.7	19.7
Mauritania	39.1	34.5	43.6
Mauritius	27.0	23.0	30.9
Mexico	16.3	15.1	17.5
Myanmar	14.1	11.6	16.6
Namibia	36.7	31.4	41.9
Nepal	40.9	37.6	44.3
Pakistan	23.0	19.6	26.4
Paraguay	19.2	16.5	22.0
Philippines	41.2	37.2	45.1
Russian Federation	47.4	42.7	52.2
South Africa	42.2	35.9	48.5
Sri Lanka	27.2	23.8	30.5
Swaziland	42.9	35.9	49.8
Tunisia	32.9	29.2	36.6
Ukraine	36.9	32.1	41.8
Uruguay	10.0	7.0	13.0
Viet Nam	15.1	10.6	19.6
Zambia	27.1	21.8	32.4
Zimbabwe	31.4	27.0	35.7

All numbers are in percentage

Tables 2 and 3 show crude prevalence of disability in the pooled study population according to socio-demographic factors, and adjusted associations between disability and socio-demographic determinants, respectively. Online Resource Tables 2–7 provide crude prevalence of disability in 43 study countries, according to each studied socio-demographic factor: sex, age, marital status, place of residence, education level, and household wealth quintile.

**Table 2:** Crude prevalence of disability among adults aged 50 years or above, by socio-demographic determinants, pooled data of 43 countries, World Health Survey, 2002–2004

	Mean	95 % CI	
Sex			
Male	24.5	23.2	25.8
Female	40.9	39.2	42.5
Age			
50–59 years	22.8	21.5	24.1
60–69 years	35.7	33.7	37.7
70–79 years	48.6	46.2	51.1
80+ years	63.2	59.7	66.8
Marital status			
Married/cohabiting	28.1	26.9	29.3
Never married	31.3	26.9	35.7
Divorced/separated/widowed	47.4	45.2	49.6
Education			
Less than primary school	40.2	38.5	41.8
Primary/secondary school completed	27.3	25.7	29.0
High school completed or above	23.5	21.2	25.9
Household economic status			
Lowest quintile	43.7	41.4	46.0
Second quintile	36.2	33.9	38.5
Middle quintile	35.9	33.5	38.2
Forth quintile	29.3	26.9	31.6
Highest quintile	21.6	19.6	23.6
Urban–rural residence			
Rural area	35.3	33.8	36.9
Urban area	30.7	29.1	32.3

All numbers are in percentage

**Table 3:** Adjusted associations between disability and the socio-demographic determinants among adults aged 50 years or above, pooled data of 43 countries, World Health Survey, 2002–2004

	Adjusted prevalence ratio^a^	95 % CI	
Sex (reference category: males)	1.51	1.42	1.60
Age (reference category: 50–59 years)			
60–69 years	1.45	1.35	1.55
70–79 years	1.88	1.75	2.02
80+ years	2.33	2.16	2.51
Marital status (reference category: married/cohabiting)			
Never married	1.03	0.90	1.17
Divorced/separated/widow	1.09	1.03	1.16
Education (reference category: high school completed or above)			
Less than primary school	1.62	1.45	1.82
Primary/secondary school completed	1.26	1.13	1.40
Household economic status (reference category: highest quintile)			
Lowest quintile	1.43	1.29	1.59
Second quintile	1.30	1.17	1.45
Middle quintile	1.35	1.22	1.50
Forth quintile	1.16	1.04	1.29
Urban–rural residence (reference category: rural area)	1.04	0.97	1.12

^a^The estimates are also adjusted for country of residence

### Prevalence according to sex

In the pooled dataset, two out of five females reported disability (unadjusted data; Table 2). The point estimate of disability was higher in females than in males in all study countries except Czech Republic; this sex difference was statistically significant in 32 countries (Online Resource Table 2).

According to the Poisson regression analysis, disability in females was 1.5 times as common as in males, controlling for other demographic and socioeconomic factors (Table 3).

### Prevalence according to age

Disability prevalence demonstrated a positive association with age. While one out of five respondents aged 50–59 years had a disability, three out of five adults aged 80+ reported disability (unadjusted data; Table 2). Differences among age groups were found to be statistically significant in all countries, however, the spread between age groups varied across countries. Sri Lanka followed by Bosnia Herzegovina, Russian Federation and Latvia showed the highest absolute difference in disability between age strata of 50–59 and 80+. For instance, less than 15 % of adults aged 50–59 in Sri Lanka reported disability, compared with over 80 % of those aged 80+ (Online Resource Table 3).

In the pooled dataset, prevalence increased significantly with each successively older age group, after controlling for covariates (Table 3).

### Prevalence according to marital status

Almost half of divorced/separated/widowed respondents in the pooled dataset reported disability, which was significantly higher than those married/cohabiting or never married (unadjusted data; Table 2). At the country level, the point estimate of disability prevalence was higher among the divorced/separated/widowed adults than among the married/cohabiting respondents in all study countries but Uruguay, and this difference was statistically significant in 36 countries (Online Resource Table 4).

### Prevalence according to place of residence

In the pooled sample, disability prevalence was higher in rural than urban areas (35.3 and 30.7 %, respectively; Table 2), although the difference was not statistically significant after taking into account covariates (Table 3). For nine countries, rural areas noted statistically significantly higher disability rates than urban areas (Online Resource Table 5).

### Prevalence according to education

Overall, the prevalence of disability was inversely associated with educational level. Those with less than primary school reported 1.6 times more disability than those with high school completed or above after controlling for other covariates (Table 3).

In three countries disability was reported only for the lowest level of education due to small sample sizes of the other two levels, and thus no comparison was made between education groups. In 32 countries disability prevalence was significantly higher in the least educated. Comparing between countries, within each category of education there was a large variation in disability prevalence. The proportion of people with less than primary education who lived with a disability ranged from 12.9 % in Malaysia to 78.1 % in Georgia. In 10 out of 40 countries with reported results, at least half of the older population with less than primary education lived with disability (Online Resource Table 6).

### Prevalence according to household economic status

The prevalence of disability increased with decreasing household economic status in the pooled sample, after controlling for confounders (Table 3).

One out of five in the richest quintile, and two out of five in the poorest quintile reported disability (unadjusted data; Table 2). In 30 countries there was a statistically significant difference across quintiles—disability was less prevalent in the richer quintiles than in the poorer quintiles. The greatest disparity was noted in Croatia—with a difference of 41.2 % points between the richest and poorest groups—followed by South Africa, Tunisia and Namibia (Online Resource Table 7).

## Discussion

This study used comparable international data to quantify disability prevalence and socio-demographic correlates within a large sample of LMICs. The overall prevalence reported in the pooled sample suggested disability rates of 33 %, comparable to a number of other studies. For example, the 2005 Survey of Income and Program Participation in the United States reported disability prevalence of 23.9 % among adults aged 45–64, and 51.8 % among those ≥65 years (Brault et al. 2009). In Malaysia, 20 % of adults over the age of 60 had functional limitations (Hairi et al. 2010), and in Brazil 23.75 % of adults 60 or older reported disability (difficulty or inability to walk 100 m) (Parahyba et al. 2009). In Spain, the rates of mild, moderate and severe/extreme disability in adults aged 75 and older were 39.17, 15.31 and 10.14 %, respectively (Virues-Ortega et al. 2011). Direct comparison of our findings with previous studies should be undertaken with caution, as different measurement criteria, data collection methods, study populations and geographical parameters can greatly affect outcomes.

Study results demonstrated a wide range of prevalence values across countries, spanning 60 % points between Malaysia and Comoros. Interestingly, countries at either extreme were not concentrated in a particular geographical region. Data indicating cross-national differences in disability prevalence have been reported across populations in the Caribbean (Schmid et al. 2008) and international censuses and surveys (Barbotte et al. 2001; Mont 2007). Elevated disability was reported in the sub-Saharan Africa region in comparison to other world regions (Murray and Lopez 1997; World Health Organization 2008). Country-specific analyses (as presented in Table 1; Online Resources Tables 2–7), however, showed significant variation within countries of this region, suggesting a role for between-country diversity stemming from, for example, differences in physical, social, political, and/or attitudinal environments (World Health Organization 2011). While our study used individual- and household-level data, subsequent ecological studies may incorporate country-level data such as gross domestic product or relevant national policies to explore their association with disability. An additional step would be to conduct a multi-level study to quantify the contributions of country-, household- and individual-level variables.

We also note the possibility that patterns of disability within a given country may be related to how that country is experiencing demographic and/or epidemiological transitions. For example, countries with decreased rates of infectious disease and an increased proportion of elderly population may demonstrate a higher prevalence of disability in older adults due to an increased number of children and younger adults in mediocre or poor health surviving to old age. Conversely, populations with fewer older adults may reflect a type of selection bias (“healthy survivor bias”), whereby only the healthiest individuals live to old age (Delgado-Rodriguez and Llorca 2004). Within countries, access to technology and support services may also impact the distribution disability prevalence among social groups. A condition that may be reported as a disability in one social group may be less limiting to those that have access to appropriate technologies and support. Systematic reporting biases also occur across different age groups and may contribute to variations in the prevalence of reported disability (Salomon et al. 2004). Variations in these reporting biases within and across countries may also contribute to variations in the estimated prevalence of disability across countries. Future studies are needed to address these possibilities in greater detail and tease apart possible measurement issues from other determinants.

Female sex was associated with higher disability prevalence, a trend which has been widely reported across epidemiological studies (World Health Organization 2011). Within LMICs, disability in older women has been attributed to non-communicable diseases, injuries, violence targeting women, limited access to health services, and poor working or living conditions (World Health Organization 2009). The years of life spent living with a disability has been estimated to be twice as long in women, who have longer life expectancies (Newman and Brach 2001). On the other hand, there have also been reports of similar disability rates between sexes or male-favourable situations (Grundy and Glaser 2000), as a minority of our country-specific results indicated.

Although disability prevalence was 33.3 % in the pooled sample of LMICs, divorced/separated/widowed respondents reported 47.4 % disability. In a similar manner, a study from Turkey also reported disability to be more prevalent among divorced/widowed/separated (19.4 %) than married or single respondents (5.4 %) (Kisioglu et al. 2003). The sequence of disability onset and divorce/separation/death is unknown in both studies. It has been suggested that people living with a disability are less likely to marry, and that disability can have negative consequences on many aspects of family life (World Health Organization 1981).

The pooled results of the 43 LMICs indicated that place of residence was non-significant after adjusting for confounding factors. Rural and urban environments each contain unique situational factors for disabilities, including greater risk of injury from accidents in urban areas (Elwan 1999), and more limited access to appropriate health treatment and services in rural areas (World Health Organization 2011).

The present study observed socioeconomic disparity in disability prevalence according to both education and household economic status. Similar trends according to education (Beydoun and Popkin 2005; Jagger et al. 2007) and economic status (Parahyba et al. 2009) have been reported in numerous samples. A study from the United States reported evidence to suggest that education- and income-based disparities in disability prevalence have widened over the past two decades (Schoeni et al. 2005). Due to the cross-sectional nature of our data, causal inferences cannot be made with certainty. Previous reports have explored a bidirectional link between disability and poverty, noting that each increases the risk for the other (Elwan 1999; World Health Organization 2011).

The issue of how to measure socioeconomic status in older adults from LMICs adds complexity to multi-country disability research. The population of adults over 50 in LMICs represents an emergent and understudied demographic within the global population. Even within higher income settings, standard indicators of socioeconomic status, including individual income, social class, education, and housing tenure may lack applicability in older populations (Matthews et al. 2005). For example, a social class gradient in functional limitation was found to persist from ages 55–84, but not in adults aged 85 and older (Minkler et al. 2006). In the United Kingdom, a subjective measure of self-perceived financial adequacy performed better than objective measures in explaining the variation and onset of disability until the age of 85 (Matthews et al. 2005), although the validity of this methodology for multinational comparisons is unknown.

The present study used available objective socioeconomic information collected through the WHS, enhancing data comparability across LMICs. The direction and magnitude of inequality in education and household wealth in the pooled sample were not different; however, individual countries demonstrated some variance. For example, Myanmar demonstrated significant differences in disability prevalence by education, but not between the richest and poorest wealth quintiles.

The results from our study point to the importance of monitoring not only average levels of disability in a given population over time, but also data that are systematically disaggregated according to relevant socio-demographic factors. In this manner, the needs of the most disadvantaged can be addressed in a targeted way. The marked gap in prevalence between the rich and poor, observed across almost all study countries, suggests that the economically disadvantaged need social protection mechanisms that address them specifically as well as health interventions that are likely to prevent disability. Similarly, other populations such as those living in rural areas and women should be provided targeted access to services as well. Unless data are collected and disaggregated systematically it will not be possible to monitor whether health and social policies are benefitting the most needy. The CRPD specifically calls on countries to gather data of this nature over time to monitor the implementation of the Convention.

### Strengths and weaknesses and implications

Limitations and uncertainties associated with the WHS methodology, disability thresholds and data analysis were previously discussed (World Health Organization 2011). Briefly, the use of self-reported data introduces uncertainty about subjective interpretation of the questions, influenced by the respondents’ understanding of the question, as well as their experiences, expectations and culture. Though we used an item response theory-based method to compute the disability score, we still note substantial differences in disability prevalence between countries that beg explanation and this raises the possibility that systematic reporting bias may have occurred due to factors not measured in our study. The validation of self-reported WHS data with comprehensive biomedical assessments and expert opinions would be optimal, albeit resource intensive.

A limitation of using cross-sectional data precludes drawing conclusions about cause and effect relationships. While certain variables are inherently independent (sex and age), others (for example, household economic status and education level) may demonstrate bidirectional relationships with disability. For example, while those with lower levels of wealth or education may be more likely to develop poor health or disability, poor health status (i.e. disability) itself may also lead to lower wealth or education attainment. The latter may be more likely to prevail in environments that offer fewer channels of assistance through health care provision or social programs. This distinction has important implications, especially within LMICs, as reforms to social policies and institutional practices may mediate the impact and development of disability.

A threshold score was established along a continuum of functioning to define disability and non-disability. The choice of threshold concurred with previous analyses of WHS data (World Health Organization 2011). Respondents with scores above the threshold experienced significant disability or functional decrements in everyday life, including conditions such as arthritis, angina, low vision or alcohol dependence (World Health Organization 2011). It is possible that the sample included false-positives and false-negatives, although the inclusion of eight health domains provided a holistic assessment of functional ability. A binary classification allowed for straightforward analyses according to socio-demographic variables; however, adopting additional thresholds to distinguish disability severity would provide a more nuanced representation of how disability affects societies (Mont 2007; World Health Organization 2011).

The use of disaggregated data was an important strength of this study, as such data helped to identify and quantify the barriers faced by people living with disabilities in 43 LMICs (United Nations 2006). The outcomes of this study are relevant to prevention and management efforts, and can be used as a basis for equity-based monitoring and evaluative approaches. Community-based rehabilitation, for example, promotes the social inclusion of people with disabilities by taking actions that specifically aim to address inequalities, uphold human rights and reduce poverty (World Health Organization 2004). Governments and stakeholders at national, intermediate and local levels have a key role in the implementation and sustainability of effective policies and programs; adopting an equity focus for monitoring and surveillance activities can help to ensure that interventions are benefiting vulnerable populations (Commission on Social Determinants of Health 2008).

### Conclusions

These findings substantiate disability as a pressing issue in older adult populations across LMICs, and provide a benchmark for tracking disability trends. To our knowledge, this is the most recent comparable, country-specific analysis of disability prevalence in adults over 50 of LMICs. Measuring disability by socio-demographic factors revealed disparity within all studied variables. In the pooled sample, disability prevalence was higher among females, those in older age brackets and divorced/separated/widowed; education and wealth levels were inversely associated with disability rates, and urban residence showed a tendency to be advantageous over rural residence. However, several of the country-specific data presented in the current study demonstrated disparate patterns, highlighting the importance of both pooled and country-specific analysis. Effective approaches aimed at disability prevention and improved disability management are warranted, such as the inclusion of equity considerations in monitoring and evaluation activities (i.e. monitoring and evaluation of disability prevalence and outcomes by socio-demographic variables).

## Electronic supplementary material

Below is the link to the electronic supplementary material.

Supplementary material 1 (PDF 350 kb)
