# Supplementary material for: Socio-demographic patterns of disability among older adult populations of low-income and middle-income countries: results from World Health Survey
Source: Int J Public Health. 2015 Nov 4;61:337–45. doi: 10.1007/s00038-015-0742-3 (PMC4879166; doi:10.1007/s00038-015-0742-3)
Supplement: Supplementary file 1 — Supplementary material 1 (PDF 350 kb) [file 38_2015_742_MOESM1_ESM.pdf]

Article Title: Socio-demographic patterns of disability among older adult populations of low-income and middle-income countries: results from World Health Survey
Journal Name: International Journal of Public Health
Author Names: Ahmad Reza Hosseinpoor, Nicole Bergen, Nenad Kostanjsek, Paul Kowal, Alana Officer, Somnath Chatterji
Corresponding Author: Ahmad Reza Hosseinpoor, Department of Health Statistics and Information Systems, World Health Organization, hosseinpoora@who.int

Online Resource Table 1
Study sample size, by country, World Health Survey, 2002-2004

	**Sample Size**		
**Country**	**Initial**	**Final**	**Missing Response**
Bangladesh	1,208	1,160	4%
Bosnia and Herzegovina	440	403	8%
Brazil	1,492	1,331	11%
Burkina Faso	917	859	6%
Chad	1,020	930	9%
China	1,409	1,300	8%
Comoros	622	575	8%
Congo	428	376	12%
Cote d'Ivoire	530	471	11%
Croatia	561	504	10%
Czech Republic	434	388	11%
Dominican Republic	1,355	1,231	9%
Ecuador	1,279	1,068	16%
Estonia	513	472	8%
Ethiopia	1,077	1,038	4%
Georgia	1,292	1,178	9%
Ghana	1,140	1,028	10%
India	2,559	2,371	7%
Kazakhstan	1,109	1,052	5%
Kenya	970	879	9%
Latvia	470	431	8%
Malawi	1,143	1,014	11%
Malaysia	1,652	1,439	13%
Mali	1,190	981	18%
Mauritania	972	806	17%
Mauritius	1,068	959	10%
Mexico	11,009	10,168	8%
Myanmar	1,606	1,524	5%
Namibia	877	780	11%
Nepal	2,117	1,783	16%
Pakistan	1,352	1,244	8%
Paraguay	1,321	1,230	7%
Philippines	2,230	2,067	7%
Russian Federation	2,308	2,103	9%
South Africa	458	404	12%
Sri Lanka	1,906	1,780	7%
Swaziland	740	629	15%
Tunisia	1,393	1,260	10%
Ukraine	1,279	1,011	21%
Uruguay	1,182	1,060	10%
Viet Nam	764	710	7%
Zambia	704	639	9%
Zimbabwe	900	811	10%
**Total**	**58,996**	**53,447**	

Online Resource Table 2
Crude prevalence of disability among adults aged 50 years or above, by sex, World Health Survey, 2002-2004

	Men		Women	
**Country**	**Estimate**	**SE**	**Estimate**	**SE**
Bangladesh*	**34.7**	2.5	**70.1**	2.7
Bosnia and Herzegovina*	**29.2**	5.3	**47.4**	5.8
Brazil*	**22.1**	1.8	**41.4**	2.1
Burkina Faso*	**20.2**	2.7	**35.2**	4.0
Chad*	**26.2**	2.7	**48.8**	3.4
China	**12.1**	2.3	**15.5**	3.8
Comoros*	**59.5**	3.4	**72.2**	3.6
Congo*	**22.5**	5.8	**42.9**	7.9
Cote d'Ivoire	**31.9**	3.7	**37.8**	4.1
Croatia	**27.3**	3.5	**35.4**	3.7
Czech Republic	**30.4**	6.4	**28.5**	3.9
Dominican Republic*	**11.9**	2.5	**24.0**	2.5
Ecuador*	**21.0**	3.5	**34.7**	2.8
Estonia	**20.0**	3.7	**24.9**	3.1
Ethiopia*	**21.6**	1.9	**34.0**	2.9
Georgia*	**30.8**	3.2	**43.9**	2.5
Ghana*	**21.6**	2.2	**29.9**	2.5
India*	**35.5**	2.7	**53.0**	3.1
Kazakhstan*	**21.4**	2.9	**47.3**	4.7
Kenya*	**25.0**	4.6	**37.6**	3.8
Latvia	**33.1**	4.8	**42.1**	3.7
Malawi*	**16.9**	2.1	**25.7**	2.5
Malaysia*	**5.0**	1.0	**10.4**	1.4
Mali	**13.6**	1.7	**18.9**	2.3
Mauritania	**34.0**	3.6	**43.4**	3.2
Mauritius*	**17.0**	2.1	**35.3**	2.9
Mexico*	**12.7**	0.7	**19.4**	0.9
Myanmar*	**11.0**	1.3	**16.7**	1.9
Namibia*	**26.3**	2.9	**44.4**	3.7
Nepal*	**32.6**	2.0	**48.9**	2.3
Pakistan*	**14.0**	1.7	**31.4**	2.8
Paraguay*	**7.4**	1.1	**30.5**	2.3
Philippines	**39.1**	2.5	**43.1**	2.4
Russian Federation*	**35.8**	3.6	**52.9**	2.8
South Africa*	**33.7**	4.8	**48.8**	3.8
Sri Lanka*	**18.4**	2.1	**36.7**	2.3
Swaziland*	**33.4**	4.9	**50.6**	4.1
Tunisia*	**23.2**	2.3	**42.0**	2.6
Ukraine*	**24.6**	3.2	**45.2**	3.2
Uruguay*	**7.2**	2.1	**12.1**	1.7
Viet Nam*	**10.7**	2.4	**18.5**	3.0
Zambia	**23.7**	3.4	**29.8**	3.2
Zimbabwe	**27.8**	2.9	**34.7**	2.8

* Statistically significant difference (P-value<0.05)
† All numbers are in percentage

Online Resource Table 3
Crude prevalence of disability among adults aged 50 years or above, by age group, World Health Survey, 2002-2004

	50-59 Years		60-69 Years		70-79 Years		80+ Years	
**Country**	**Estimate**	**SE**	**Estimate**	**SE**	**Estimate**	**SE**	**Estimate**	**SE**
Bangladesh*	**42.7**	2.8	**55.6**	3.4	**71.5**	4.6	**87.7**	4.6
Bosnia and Herzegovina*	**16.1**	4.7	**46.7**	6.7	**59.2**	8.7	**-**	-
Brazil*	**26.8**	2.2	**35.0**	2.7	**38.2**	3.5	**58.7**	6.0
Burkina Faso*	**15.9**	3.1	**34.1**	4.4	**47.8**	6.8	**62.9**	11.3
Chad*	**30.3**	3.5	**46.1**	4.1	**45.0**	4.9	**59.2**	6.6
China*	**6.4**	2.1	**11.8**	2.9	**26.3**	5.5	**47.6**	9.0
Comoros*	**59.9**	4.0	**65.0**	4.5	**75.1**	5.0	**(78.4)**	7.6
Congo*	**32.7**	8.1	**21.8**	6.9	**62.7**	9.0	**-**	-
Cote d'Ivoire*	**24.2**	3.9	**35.9**	5.1	**59.7**	7.4	**(79.2)**	10.1
Croatia*	**21.0**	3.6	**28.5**	4.1	**52.0**	5.6	**(66.1)**	9.0
Czech Republic*	**17.9**	4.5	**18.0**	5.0	**52.7**	7.7	**(61.7)**	15.5
Dominican Republic*	**12.0**	2.2	**18.5**	3.1	**29.2**	6.0	**42.1**	7.5
Ecuador*	**21.2**	3.5	**23.7**	3.4	**41.2**	6.0	**57.1**	10.4
Estonia*	**16.8**	3.0	**16.6**	2.9	**33.9**	6.5	**(43.1)**	8.3
Ethiopia*	**19.3**	2.0	**35.4**	3.3	**39.0**	5.0	**50.6**	9.2
Georgia*	**21.2**	3.3	**34.6**	2.9	**57.4**	5.0	**78.9**	6.9
Ghana*	**17.0**	2.1	**27.2**	3.1	**44.8**	4.8	**64.3**	5.3
India*	**32.2**	2.6	**49.8**	3.2	**61.5**	3.9	**71.3**	6.1
Kazakhstan*	**15.6**	2.2	**38.4**	5.1	**75.6**	4.7	**-**	-
Kenya*	**21.5**	4.2	**36.2**	4.6	**48.0**	5.7	**59.3**	8.4
Latvia*	**21.9**	4.2	**31.7**	4.8	**58.6**	4.9	**(83.4)**	9.2
Malawi*	**15.3**	2.2	**21.0**	3.3	**29.7**	3.7	**64.1**	8.3
Malaysia*	**3.4**	0.9	**8.5**	1.8	**16.3**	3.7	**31.4**	6.6
Mali*	**11.3**	2.0	**17.7**	2.6	**31.5**	4.9	**37.0**	5.8
Mauritania*	**31.3**	3.3	**42.3**	3.8	**56.0**	5.7	**(56.4)**	12.1
Mauritius*	**20.5**	2.4	**23.2**	2.8	**41.0**	4.6	**61.1**	6.6
Mexico*	**9.4**	0.6	**15.2**	1.0	**27.6**	1.5	**41.2**	2.7
Myanmar*	**5.4**	1.2	**12.2**	1.5	**32.0**	3.6	**51.5**	7.6
Namibia*	**27.5**	3.5	**32.9**	4.1	**59.9**	5.8	**60.9**	6.2
Nepal*	**30.2**	2.2	**46.2**	2.6	**56.8**	3.1	**79.0**	5.4
Pakistan*	**14.7**	1.8	**26.8**	3.6	**38.3**	4.5	**52.5**	6.2
Paraguay*	**16.5**	1.8	**17.7**	2.5	**23.9**	3.5	**39.9**	7.8
Philippines*	**34.5**	2.4	**40.8**	2.7	**58.1**	4.3	**73.1**	6.3
Russian Federation*	**20.1**	2.5	**43.6**	4.6	**62.6**	4.4	**85.8**	3.3
South Africa*	**37.4**	4.6	**41.5**	5.2	**48.1**	7.3	**-**	-
Sri Lanka*	**12.8**	1.4	**29.7**	2.9	**47.7**	4.9	**81.1**	4.9
Swaziland*	**30.2**	4.1	**52.0**	5.3	**55.6**	6.4	**66.9**	7.5
Tunisia*	**20.9**	2.3	**33.9**	3.2	**50.7**	3.7	**69.8**	5.2
Ukraine*	**20.2**	2.6	**38.4**	4.0	**50.9**	4.5	**67.4**	8.3
Uruguay*	**6.7**	1.3	**10.4**	3.2	**9.6**	2.7	**18.6**	3.7
Viet Nam*	**6.3**	1.5	**14.6**	2.9	**28.4**	5.8	**-**	-
Zambia*	**18.1**	3.3	**26.6**	3.6	**42.2**	5.1	**(74.3)**	10.9
Zimbabwe*	**22.9**	2.9	**31.5**	3.6	**41.9**	6.1	**76.7**	6.5

* Statistically significant difference (P-value<0.05)
† All numbers are in percentage
†† The figures in the parentheses are based on small numbers of samples (25-49 unweighted samples)
††† The estimates are not shown due to very small number of samples (<25 unweighted samples)

Online Resource Table 4
Crude prevalence of disability among adults aged 50 years or above, by marital status, World Health Survey, 2002-2004

	Married/Cohabiting		Never Married		Divorced/Separated/Widowed	
**Country**	**Estimate**	**SE**	**Estimate**	**SE**	**Estimate**	**SE**
Bangladesh*	**42.5**	2.4	**-**	-	**78.1**	2.8
Bosnia and Herzegovina*	**31.1**	5.1	**-**	-	**60.3**	6.1
Brazil*	**29.2**	1.8	**28.9**	5.5	**42.2**	2.8
Burkina Faso*	**23.7**	2.9	**-**	-	**49.2**	6.2
Chad*	**32.9**	3.2	**-**	-	**50.3**	3.5
China*	**11.0**	2.5	**-**	-	**29.0**	6.0
Comoros	**62.9**	3.2	**-**	-	**73.4**	4.3
Congo*	**22.8**	7.1	**(67.5)**	11.8	**48.9**	9.1
Cote d'Ivoire*	**30.5**	3.7	**(25.5)**	9.3	**44.0**	5.4
Croatia*	**27.1**	3.2	**(42.9)**	13.7	**44.4**	4.1
Czech Republic	**25.7**	4.6	**-**	-	**38.4**	5.2
Dominican Republic*	**15.0**	2.5	**14.5**	6.0	**24.6**	2.7
Ecuador*	**24.5**	3.0	**29.1**	6.2	**37.6**	3.8
Estonia*	**15.8**	3.8	**(14.3)**	5.4	**33.3**	3.8
Ethiopia*	**25.2**	2.0	**-**	-	**34.9**	3.5
Georgia*	**31.3**	2.8	**34.1**	5.1	**56.0**	3.6
Ghana*	**21.2**	2.0	**(38.4)**	15.3	**35.8**	3.0
India*	**39.5**	2.1	**(44.7)**	11.6	**61.7**	4.2
Kazakhstan*	**24.5**	2.6	**(34.0)**	9.9	**58.0**	6.2
Kenya	**29.5**	3.5	**-**	-	**37.7**	5.0
Latvia	**33.3**	4.0	**(37.6)**	9.8	**43.6**	4.3
Malawi*	**18.2**	2.3	**-**	-	**29.4**	3.0
Malaysia*	**5.3**	0.8	**(3.5)**	2.5	**17.8**	3.1
Mali*	**13.1**	1.6	**6.4**	3.8	**29.9**	4.2
Mauritania*	**35.1**	2.8	**(48.1)**	15.5	**48.2**	4.1
Mauritius*	**19.9**	1.8	**27.8**	8.2	**43.0**	3.6
Mexico*	**13.5**	0.6	**15.1**	2.1	**24.2**	1.3
Myanmar*	**10.7**	1.2	**11.3**	3.7	**23.6**	2.8
Namibia*	**32.9**	3.4	**33.3**	4.9	**47.0**	5.0
Nepal*	**37.4**	1.8	**-**	-	**53.5**	3.0
Pakistan*	**19.5**	1.9	**(23.6)**	13.0	**41.2**	4.6
Paraguay*	**16.9**	1.5	**17.7**	3.3	**29.0**	3.9
Philippines*	**38.1**	2.1	**47.8**	6.5	**50.2**	3.4
Russian Federation*	**38.2**	3.2	**52.5**	8.7	**54.4**	3.4
South Africa	**39.1**	4.3	**44.4**	6.6	**46.8**	6.0
Sri Lanka*	**23.0**	2.0	**22.3**	4.9	**44.1**	3.2
Swaziland	**40.9**	3.6	**43.2**	8.2	**51.4**	7.6
Tunisia*	**29.1**	2.0	**(24.3)**	9.6	**53.5**	3.6
Ukraine*	**30.1**	2.8	**(48.4)**	10.0	**48.4**	3.9
Uruguay	**9.5**	1.9	**19.6**	4.2	**9.2**	1.7
Viet Nam*	**12.0**	2.1	**-**	-	**27.7**	6.1
Zambia*	**22.3**	3.1	**-**	-	**38.7**	4.1
Zimbabwe*	**28.2**	2.6	**-**	-	**39.4**	3.8

* Statistically significant difference between married/cohabiting and divorced/separated/widowed groups (P-value<0.05)
† All numbers are in percentage
†† The figures in the parentheses are based on small numbers of samples (25-49 unweighted samples)
††† The estimates are not shown due to very small number of samples (<25 unweighted samples)

Online Resource Table 5
Crude prevalence of disability among adults aged 50 years or above, by place of residence, World Health Survey, 2002-2004

	Rural		Urban	
**Country**	**Estimate**	**SE**	**Estimate**	**SE**
Bangladesh*	**55.5**	2.4	**38.6**	3.6
Bosnia and Herzegovina	**40.2**	6.2	**37.8**	7.1
Brazil	**37.0**	4.1	**31.7**	1.7
Burkina Faso	**28.6**	3.3	**33.0**	4.3
Chad	**38.3**	2.9	**39.1**	5.3
China	**11.8**	3.8	**17.4**	2.6
Comoros	**67.8**	3.3	**62.5**	4.3
Congo	**41.8**	10.3	**31.8**	5.4
Cote d'Ivoire	**29.0**	3.9	**37.4**	4.0
Croatia	**36.0**	4.6	**30.4**	3.2
Czech Republic	**31.0**	6.2	**28.6**	4.1
Dominican Republic	**18.3**	2.9	**18.1**	2.1
Ecuador	**29.9**	3.2	**26.0**	3.5
Estonia	**23.7**	3.8	**22.6**	3.5
Ethiopia	**28.8**	2.0	**24.1**	5.6
Georgia	**40.7**	3.9	**35.9**	2.7
Ghana	**26.7**	2.2	**25.3**	2.6
India	**44.4**	1.8	**41.3**	3.0
Kazakhstan	**32.4**	4.5	**38.5**	5.6
Kenya	**34.4**	2.5	**18.1**	11.2
Latvia	**42.9**	4.7	**36.5**	3.8
Malawi	**21.8**	1.9	**17.7**	6.3
Malaysia*	**11.5**	1.9	**5.0**	0.9
Mali	**17.0**	1.8	**16.0**	3.0
Mauritania	**43.2**	3.1	**35.5**	3.4
Mauritius*	**32.1**	3.0	**21.5**	2.3
Mexico*	**18.6**	1.1	**15.5**	0.7
Myanmar	**13.7**	1.4	**15.0**	2.7
Namibia	**38.7**	3.1	**29.1**	4.9
Nepal	**42.1**	1.8	**32.0**	4.5
Pakistan	**21.5**	2.0	**26.1**	3.5
Paraguay*	**23.1**	1.9	**16.4**	2.0
Philippines*	**49.6**	2.9	**34.9**	2.6
Russian Federation	**53.6**	4.3	**46.5**	2.7
South Africa*	**53.2**	4.1	**31.7**	4.5
Sri Lanka*	**29.6**	1.7	**18.3**	2.8
Swaziland	**43.9**	4.0	**37.5**	7.8
Tunisia*	**39.3**	3.1	**28.7**	2.2
Ukraine	**36.2**	5.3	**37.3**	2.5
Uruguay	**7.8**	2.0	**10.2**	1.7
Viet Nam	**16.1**	2.8	**12.2**	3.9
Zambia	**29.3**	3.3	**19.9**	3.8
Zimbabwe	**30.5**	2.4	**34.4**	5.3

* Statistically significant difference (P-value<0.05)
† All numbers are in percentage

Online Resource Table 6
Crude prevalence of disability among adults aged 50 years or above, by level of education, World Health Survey, 2002-2004

	**Less than Primary School**		**Primary/Secondary School Completed**		**High School Completed or Above**	
**Country**	**Estimate**	**SE**	**Estimate**	**SE**	**Estimate**	**SE**
Bangladesh*	**59.7**	2.4	**31.6**	4.0	**16.6**	6.8
Bosnia and Herzegovina	**46.2**	7.1	**37.6**	5.2	**(8.4)**	7.2
Brazil*	**42.8**	2.0	**23.9**	2.5	**20.0**	3.7
Burkina Faso	**29.2**	2.8	-	-	-	-
Chad*	**40.1**	2.6	**(4.0)**	3.0	-	-
China*	**19.6**	4.6	**10.7**	3.3	**8.1**	2.2
Comoros	**67.3**	2.7	-	-	-	-
Congo*	**45.0**	5.9	**23.8**	11.8	**(7.9)**	5.1
Cote d'Ivoire	**38.3**	3.2	**21.9**	5.8	-	-
Croatia*	**52.5**	5.0	**28.5**	3.3	**12.8**	4.4
Czech Republic*	-	-	**36.2**	4.9	**18.2**	4.6
Dominican Republic	**19.2**	2.2	**12.4**	3.5	**(13.9)**	6.8
Ecuador*	**39.1**	3.7	**22.0**	2.7	**4.9**	2.6
Estonia*	**38.8**	6.2	**28.8**	5.1	**17.7**	3.1
Ethiopia*	**30.4**	2.0	**5.9**	2.9	-	-
Georgia*	**78.1**	6.9	**56.0**	6.0	**33.7**	2.3
Ghana	**28.7**	2.1	**22.6**	2.8	**(18.1)**	6.7
India*	**50.6**	2.0	**34.3**	3.4	**15.2**	3.0
Kazakhstan*	-	-	**53.8**	6.0	**32.8**	4.4
Kenya	**36.4**	3.1	**24.0**	7.4	**14.2**	8.6
Latvia*	**66.3**	8.2	**37.2**	3.7	**34.5**	5.4
Malawi	**22.2**	1.9	**15.6**	7.1	-	-
Malaysia*	**12.9**	1.8	**4.4**	1.1	**1.0**	0.6
Mali	**17.3**	1.6	-	-	-	-
Mauritania	**40.8**	2.5	**(29.3)**	9.6	-	-
Mauritius*	**36.8**	2.8	**18.7**	2.3	**2.4**	2.4
Mexico*	-	-	**17.3**	**0.6**	**8.8**	1.4
Myanmar*	**17.4**	1.7	**8.3**	1.5	**2.8**	2.0
Namibia*	**41.9**	3.1	**21.2**	4.4	-	-
Nepal*	**43.4**	1.7	**20.7**	3.9	**(10.8)**	8.0
Pakistan*	**26.4**	2.1	**12.0**	3.5	**7.5**	2.4
Paraguay*	**21.3**	1.7	**19.3**	2.8	**5.2**	2.6
Philippines*	**55.9**	2.6	**34.3**	2.4	**23.6**	3.7
Russian Federation*	**72.9**	6.1	**57.8**	2.8	**33.7**	3.6
South Africa*	**55.0**	4.4	**33.7**	5.2	**19.6**	5.7
Sri Lanka*	**48.6**	3.9	**25.3**	1.6	**12.3**	3.5
Swaziland*	**46.1**	4.4	**41.6**	5.2	**(17.0)**	6.2
Tunisia*	**39.3**	2.3	**22.2**	4.0	**8.2**	2.6
Ukraine*	**65.0**	8.4	**51.6**	4.8	**30.5**	2.6
Uruguay	**14.0**	2.9	**9.5**	1.7	**7.4**	1.4
Viet Nam*	**26.2**	4.2	**8.0**	1.8	**3.1**	1.4
Zambia*	**32.9**	3.2	**12.5**	3.4	-	-
Zimbabwe*	**36.2**	2.7	**22.5**	3.3	-	-

* Statistically significant difference (P-value<0.05)
† All numbers are in percentage
†† The figures in the parentheses are based on small numbers of samples (25-49 unweighted samples)
††† The estimates are not shown due to very small number of samples (<25 unweighted samples)

Online Resource Table 7
Crude prevalence of disability among adults aged 50 years or above, by household wealth quintile, World Health Survey, 2002-2004

	**Lowest Quintile**		**Second Quintile**		**Middle Quintile**		**Fourth Quintile**		**Highest Quintile**	
**Country**	**Estimate**	**SE**	**Estimate**	**SE**	**Estimate**	**SE**	**Estimate**	**SE**	**Estimate**	**SE**
Bangladesh*	**66.4**	4.4	**53.4**	4.5	**62.6**	4.2	**40.9**	3.9	**36.6**	4.1
Bosnia and Herzegovina*	**53.6**	6.7	**36.0**	6.9	**51.6**	9.4	**34.7**	8.6	**25.1**	8.4
Brazil*	**42.3**	3.5	**41.1**	3.6	**40.5**	3.5	**30.1**	3.0	**14.4**	2.6
Burkina Faso	**31.0**	5.0	**30.0**	4.5	**24.9**	5.0	**30.8**	5.0	**27.4**	7.2
Chad*	**48.2**	4.3	**42.9**	4.4	**31.7**	6.0	**34.5**	5.4	**29.7**	5.8
China*	**23.8**	5.9	**19.5**	2.8	**13.3**	4.3	**10.9**	3.8	**3.7**	1.8
Comoros	**71.0**	4.5	**69.9**	4.4	**61.8**	5.5	**69.1**	6.1	**59.9**	4.6
Congo	**54.0**	11.1	**40.8**	15.2	**49.7**	8.8	**17.3**	7.5	**24.1**	11.1
Cote d'Ivoire	**26.9**	4.9	**34.9**	5.2	**42.9**	6.2	**39.4**	6.0	**26.0**	7.4
Croatia*	**57.4**	4.8	**40.0**	5.0	**25.7**	4.9	**23.6**	5.4	**16.2**	5.0
Czech Republic*	**40.6**	7.2	**25.5**	7.1	**30.8**	7.2	**11.6**	5.0	**(13.7)**	7.9
Dominican Republic	**16.0**	3.8	**19.0**	3.3	**18.1**	4.7	**20.8**	5.2	**16.6**	2.8
Ecuador*	**40.4**	5.1	**27.0**	4.3	**24.3**	5.0	**27.9**	5.0	**17.4**	2.8
Estonia*	**33.6**	5.3	**31.4**	4.1	**19.1**	4.2	**9.7**	2.8	**3.1**	2.5
Ethiopia	**32.2**	3.2	**32.2**	3.5	**29.6**	3.8	**28.0**	4.3	**17.7**	4.0
Georgia*	**55.8**	4.0	**44.5**	4.4	**35.9**	4.7	**34.9**	5.2	**25.1**	2.9
Ghana*	**33.2**	3.4	**34.1**	4.0	**26.1**	3.3	**20.0**	3.2	**21.2**	3.7
India*	**52.8**	3.8	**44.2**	4.7	**45.5**	3.3	**45.8**	5.1	**32.6**	3.5
Kazakhstan*	**45.9**	4.5	**40.4**	4.7	**39.2**	9.4	**36.8**	5.3	**18.2**	5.0
Kenya*	**44.9**	5.0	**46.4**	6.5	**28.3**	5.1	**17.6**	5.6	**26.5**	7.8
Latvia*	**46.5**	5.1	**37.5**	6.3	**49.6**	6.9	**24.2**	5.4	**26.5**	6.6
Malawi	**24.0**	2.4	**19.0**	2.7	**25.6**	4.0	**18.0**	4.5	**20.7**	5.2
Malaysia*	**18.1**	3.5	**6.1**	1.4	**6.3**	1.7	**5.6**	1.7	**4.2**	1.4
Mali	**17.6**	3.0	**15.9**	3.2	**19.7**	3.7	**19.9**	3.4	**10.6**	3.1
Mauritania	**48.2**	5.2	**44.6**	4.5	**37.3**	5.0	**37.0**	4.3	**33.2**	5.3
Mauritius*	**43.4**	3.8	**37.5**	4.3	**23.6**	3.5	**20.5**	3.5	**12.5**	2.8
Mexico*	**20.3**	1.3	**20.1**	1.3	**17.9**	1.2	**14.6**	1.2	**11.4**	1.1
Myanmar*	**16.9**	3.0	**20.3**	3.1	**12.3**	2.2	**10.6**	1.9	**12.6**	2.0
Namibia*	**51.8**	4.5	**34.5**	5.2	**36.8**	5.1	**31.4**	5.4	**18.2**	5.0
Nepal*	**47.9**	3.8	**41.1**	3.1	**38.6**	3.3	**48.1**	3.3	**30.4**	2.9
Pakistan	**25.1**	3.7	**23.0**	3.5	**25.9**	3.9	**22.4**	3.5	**17.2**	3.0
Paraguay*	**28.5**	3.6	**17.3**	2.6	**21.7**	3.0	**18.0**	2.7	**15.4**	3.0
Philippines*	**54.6**	3.0	**47.4**	4.0	**47.3**	3.4	**37.3**	3.5	**27.2**	3.3
Russian Federation*	**59.6**	3.4	**44.1**	4.8	**48.8**	4.5	**29.5**	4.2	**39.8**	10.7
South Africa*	**58.6**	6.0	**38.5**	6.4	**41.8**	6.4	**40.6**	6.5	**18.9**	5.7
Sri Lanka*	**46.7**	3.9	**38.0**	3.5	**29.8**	3.0	**26.1**	3.0	**16.5**	2.2
Swaziland*	**56.8**	5.9	**53.2**	6.6	**41.6**	6.9	**32.2**	5.4	**36.2**	6.2
Tunisia*	**50.6**	4.0	**38.1**	4.2	**31.1**	3.4	**31.6**	3.7	**15.6**	3.1
Ukraine	**46.1**	5.0	**36.7**	3.8	**36.7**	4.6	**30.4**	5.6	**29.9**	4.4
Uruguay*	**20.6**	3.6	**12.4**	3.0	**10.5**	1.8	**6.1**	0.9	**3.3**	1.5
Viet Nam*	**27.0**	7.0	**19.4**	3.9	**17.0**	4.3	**8.0**	3.1	**9.4**	3.1
Zambia	**35.6**	4.3	**26.4**	4.8	**26.3**	6.7	**23.1**	5.6	**18.0**	5.2
Zimbabwe	**38.2**	5.4	**38.1**	4.9	**31.0**	5.3	**29.8**	4.3	**22.6**	4.6

* Statistically significant difference (P-value<0.05)
† All numbers are in percentage
†† The figures in the parentheses are based on small numbers of samples (25-49 unweighted samples)
